# Novel in vitro inhibitory functions of potato tuber proteinaceous inhibitors

**DOI:** 10.1007/s00438-014-0906-5

**Published:** 2014-09-27

**Authors:** Matthias Fischer, Markus Kuckenberg, Robin Kastilan, Jost Muth, Christiane Gebhardt

**Affiliations:** 1 Department Plant Breeding and Genetics, Max-Planck Institute for Plant Breeding Research, Carl von LinnéWeg 10, 50829 Cologne, Germany; 2 Fraunhofer Institute for Molecular Biology and Applied Ecology, Forckenbeckstraße 6, 52074 Aachen, Germany

**Keywords:** Potato (*Solanum tuberosum* L.), Tuber, Enzyme inhibitor, Protease inhibitor, Heterologous expression, *Pichia pastoris*

## Abstract

Plant protease inhibitors are a structurally highly diverse and ubiquitous class of small proteins, which play various roles in plant development and defense against pests and pathogens. Particular isoforms inhibit in vitro proteases and other enzymes that are not their natural substrates, for example proteases that have roles in human diseases. Mature potato tubers are a rich source of several protease inhibitor families. Different cultivars have different inhibitor profiles. With the objective to explore the functional diversity of the natural diversity of potato protease inhibitors, we randomly selected and sequenced 9,600 cDNA clones originated from mature tubers of ten potato cultivars. Among these, 120 unique inhibitor cDNA clones were identified by homology searches. Eighty-eight inhibitors represented novel sequence variants of known plant protease inhibitor families. Most frequent were Kunitz-type inhibitors (KTI), potato protease inhibitors I and II (PIN), pectin methylesterase inhibitors, metallocarboxypeptidase inhibitors and defensins. Twenty-three inhibitors were functionally characterized after heterologous expression in the yeast *Pichia pastoris*. The purified recombinant proteins were tested for inhibitory activity on trypsin, eleven pharmacological relevant proteases and the non-proteolytic enzyme 5-lipoxygenase. Members of the KTI and PIN families inhibited pig pancreas elastase, β-Secretase, Cathepsin K, HIV-1 protease and potato 5-lipoxygenase. Our results demonstrate in vitro inhibitory diversity of small potato tuber proteins commonly known as protease inhibitors, which might have biotechnological or medical applications.

**Electronic supplementary material:**

The online version of this article (doi:10.1007/s00438-014-0906-5) contains supplementary material, which is available to authorized users.

## Introduction

Protease inhibitors (PIs) are ubiquitous, small proteins which are particularly abundant in plant reproductive and storage organs such as seeds and tubers (Ryan 1990). They account for 1–10 % of the total protein in storage tissues. In higher plants, several PI families have been characterized for their molecular structure and biochemical function. The structural basis of the interaction between proteases and their inhibitors is under continuous investigation (Bateman and James 2011; Mosolov and Valueva 2005). PIs are involved in many physiological processes via control of protease activity. The best characterized example is their role in wound-induced defense responses of plants against herbivores and pathogens (Hartl et al. 2011; Jorgensen et al. 2006). They are also considered as storage proteins (Ryan 1990).

Potato tuber protein consists mainly of patatin, the major storage protein, and numerous low molecular weight PIs which can be classified in at least ten different families (De Leo et al. 2002; Rawlings et al. 2008). Most abundant are inhibitors of serine proteases from families known as Kunitz-type inhibitors (KTI), potato protease inhibitors I and II (PIN I, PIN II) and Bowman–Birk inhibitors (BBIs). KTIs are one of the best characterized inhibitors. These vacuolar proteins are abundant in potato tubers and represent a highly diverse group of proteins (Heibges et al. 2003a). Most KTIs are encoded at a complex locus on potato chromosome III, which is linked to a quantitative trait locus (QTL) for resistance to the oomycete *Phytophthora infestans* (Heibges et al. 2003a; Odeny et al. 2010). Most KTIs consist of a single polypeptide chain of approximately 24 kDa with two disulfide bridges and a single reactive site. Depending on the cultivar studied, potato KTIs were classified in three to six structural subgroups (A, B, C, D, K and M) (Bauw et al. 2006; Heibges et al. 2003a; Ishikawa et al. 1994; Oliva et al. 2010). The tremendous structural variability among KTIs suggested functional diversity (Heibges et al. 2003b). Previous studies revealed that KTIs have distinct target specificities in vitro and some have dual or broad specificity. Inhibitors of subgroup KTI-A reduced the activity of serine or aspartic proteases such as trypsin or cathepsin D (Heibges et al. 2003b; Ishikawa et al. 1994). Members of subgroup KTI-B inhibited trypsin, chymotrypsin or elastase and members of subgroup KTI-C inhibited not only subtilisin and cysteine proteases, but also other enzymes like invertase (Glaczinski et al. 2002; Heibges et al. 2003b). Similar to KTIs, the PIN I and PIN II families display high structural and functional diversity, particularly in the Solanaceae, and are organized as gene clusters mainly on potato chromosome IX and III, respectively. Plant PINs have been characterized at the biochemical and molecular level. PIN I protein was first isolated from potato tubers (Balls and Ryan 1963). More recent studies demonstrated PIN I expression in leaves, stems, flowers and tuber sprouts, which is regulated by both environmental and developmental signals (Johnson and Ryan 1990; Turra et al. 2009; Valueva et al. 2003). Proteins homologous to PIN I are found in several plant species such as barley or maize, while PIN II’s seem to be restricted to the Solanaceae (Mosolov and Valueva 2005). PIN proteins are suggested to function in plant interactions with herbivores and microbes. Digestive enzymes in the guts of herbivores were inhibited by plant PINs, restricting the absorption of essential amino acids and consequently interfering with herbivore growth and development (Chen 2008). In vitro assays confirmed inhibitory effects of plant PINs on the digestive serine proteases trypsin, chymotrypsin or subtilisin (Hartl et al. 2010; Mosolov and Valueva 2005; Turra et al. 2009).

The detrimental effect observed on herbivores and pests led to the development of inhibitor-transgenic plants (Chen 2008; Dunse et al. 2010). However, due to the adaptation of herbivores by maintaining diverse digestive enzymes and over-expressing inhibitor insensitive enzymes, and last but not least due to the rejection of transgenic crops by the public, transgenic approaches have not been widely adopted in commercial food crops (Jongsma and Bolter 1997; Zhu et al. 2005). Beyond plant biotechnology, plant PIs became attractive targets in pharmacology and drug development. Inhibitors of KTI and BBI families, purified from different leguminous seeds, were shown to block the activity of several proteases and enzymes involved in human diseases (reviewed in (Oliva and Sampaio 2009)). Plant KTIs inhibited proteins acting in the blood clotting cascade or in fibrinolysis such as factor XIIa, factor Xa, thrombin, plasmin, plasma kallikrein or tissue plasminogen activator (Cruz-Silva et al. 2004; Oliva and Sampaio 2008; Oliva et al. 2000). Elastase and cathepsin G involved in inflammatory processes in humans were shown to be inhibited by KTIs isolated from *Bauhinia* seeds (Neuhof et al. 2003; Oliveira et al. 2010). Several studies revealed anti-tumor activity of BBI and KTI inhibitors (Oliva and Sampaio 2009; Oliva et al. 2010). Trypsin inhibitors from *Peltophorum dubium* (PDTI) and soybean induced cell death of human leukemic Jurkat cell lines by activation of caspases 3 and 8. Inhibitors from seeds of the Chinese black soybean Glycine max suppressed cell proliferation of MCF-7 breast cancer cells and HepG2 hepatoma cells. Peptides derived from *Bauhinia rufa* trypsin Inhibitor (BrTI) inhibited the adhesion of tumor cells to extracellular matrix glycoproteins (Nakahata et al. 2006). In summary, KTI effects were investigated in inflammation, thrombosis, AIDS (auto immune deficiency syndrome), parasitic diseases and antifungal activity (Oliva et al. 2010).

In this paper, we tap at the tremendous structural diversity of proteinaceous inhibitors found in tubers of different potato genotypes and their potential inhibitory functions. Novel variants of KTI, PIN I, PIN II and other types of proteinaceous inhibitors were isolated as full length cDNAs from tubers of ten different potato varieties. A subset was expressed as recombinant fusion proteins in the yeast *Pichia pastoris* and examined for inhibitory properties on serine, cysteine and aspartic proteases involved in human diseases including Alzheimer disease, AIDS and osteoporosis, as well as on the potato enzyme lipoxygenase.

## Materials and methods

### Plant material

Field grown tubers from nine potato varieties (Lady Claire, Omega, Eurobeta, Verdi, Elfe, Marabel, Allians, Solara, Melba) and one breeding clone (Breeding clone 18) were provided by EUROPLANT Pflanzenzucht GmbH (Ebstorf, Germany). Tubers were peeled, washed and snap frozen in liquid nitrogen. Tuber tissue was homogenized by Mixer Mill MM 200 (Retsch, Haan, Germany) and stored at −80 °C until use.

### cDNA libraries

Total RNA was isolated from 5 g powdered, frozen tuber tissue using the PureLink^®^ Plant-RNA reagent (Invitrogen™, Carlsbad, USA) according to the manufacturer’s guidelines. Poly A^+^ RNA was prepared from total RNA using the Poly(A)Purist™ MAG purification kit (Ambion^®^, Austin, USA) following the suppliers instructions. Ten cDNA libraries were constructed from Poly A^+^ RNA of the ten genotypes. cDNA synthesis and transformation of Electromax™ DH10B™ T1 Phage resistant *E. coli* cells were performed using to the CloneMiner™ II cDNA Library construction kit (Invitrogen™, Carlsbad, USA) and the supplier’s protocol. 960 cDNA clones per library were randomly selected (9,600 clones total) and stored in 96-well microtiter plates.

### cDNA sequence analysis

Plasmid insertions were sequenced from the 5′ end using a primer (5′GTAAAACGACGGCCAGT3′) matching to the M13-forward (−20) priming site of the pDONR™222 vector which was used for cDNA library construction. Custom DNA Sanger-sequencing was performed at the Max-Planck Genome Center [Cologne, Germany (http://mpgc.mpipz.mpg.de/home/)]. Inhibitor sequences were identified by BLAST comparisons to the NCBI nucleotide collection (NR/NT). cDNA clones coding for various inhibitors of proteases and other enzymes were selected and sequenced from the 3′ end using a primer (5′AACAGCTATGACCATG3′) specific for the M13-reverse priming site to identify full length cDNA clones. Recombinant *E. coli* clones harboring inhibitor sequences were propagated in LB-media (1 % Tryptone, 0.5 % yeast extract, 1 % NaCl, pH 7) containing 50 µg/ml kanamycin. Plasmid DNA was isolated using the Plasmid Mini Kit (QIAGEN^®^, Hilden, Germany) according to the manufacturer’s guidelines and stored at −20 °C.

### Heterologous expression of cDNAs encoding protease inhibitors

cDNAs encoding mature protease inhibitors were cloned into the multiple cloning sites (MCS) of the expression vector pPICZαA (Invitrogen™, Carlsbad, USA) without their putative signal sequences. Putative signal sequences were identified using SignalP 4.0 software (http://www.cbs.dtu.dk/services/SignalP/). Translation termination sequences were omitted in fusing inhibitor sequences with the c-myc epitope and His-tag of the pPICZαA vector. Expression of fusion proteins was controlled by the methanol-inducible AOX1 promoter. The native α-factor secretion signal sequence from *Saccharomyces cerevisiae* allowed secretion of the fusion protein into the medium. For expression cloning, inhibitor sequences were amplified by PCR using 1 ng plasmid DNA as template and the primers specified in Table 1. PCR conditions were: 95 °C for 5 min, followed by 20 cycles of 95 °C for 1 min, 60 °C for 1 min and 72 °C for 1.5 min with a final extension step at 72 °C for 5 min. Cloning of PCR products into the pPICZαA vector and transformation of *Pichia pastoris* cells (strain GS115) was accomplished with the EasySelect™ Pichia Expression Kit (Invitrogen™, Carlsbad, USA) according to the suppliers protocol. Nucleotide sequences were verified by direct sequencing.

**Table 1:** Primer sequences for expression cloning of 23 potato protease inhibitors in pPICZαA

Type	Inhibitor	Forward primer (5′–3′)^a^	Reverse primer (5′–3′)^a^
KTI-A	PI0875	AAAGAATTCATGGAATCTCCTGTACCTAAGCC	AAAGCGGCCGCGACTTCCTGGAATAAGACATCAAGA
	PI4063	AAAGAATTCATGGATACTACTCCATGTACTCCAGT	AAAGCGGCCGCGACTTCCTGGAAATAGACATCAAGA
	PI6033	AAAGAATTCATGGAATCTCCTGTACCTAAGCC	AAAGCGGCCGCGACTTCCTGGAATAAGACGTCAA
	PI9070	AAAGAATTCATGGAATCTCCTGTACCTAAGCC	AAAGCGGCCGCGACTTCCTGGAATAAGACATCAAGA
	PI8311	AAAGAATTCATGCTACCCAGTGCTAAGTCTGT	AAAGCGGCCGCGTCTTCGACTTTCTCAAATTCGA
KTI-B	PI2112	AAAGAATTCATGCCAGTACTTGACGTAACTGG	AAAGCGGCCGCCTGGACTTGCTTGAAGGAGAC
	PI2568	AAAGAATTCATGCCAGTACTTGACGTAACTGG	AAAGCGGCCGCCTGGACTTGCTTGAAGGAGAC
	PI4435	AAAGAATTCATGCTACCTAGTGATGCTACTCCAGTACTT	AAAGCGGCCGCCTGGACTTGCATGAAGGAGAC
	PI4587	AAAGAATTCATGCTACCTAGTGATGCTACTCCAGTACTT	AAAGCGGCCGCCTGGACTTGCTTGAAGGAGAC
	PI5887	AAAGAATTCATGCCAGTACTTGACGTAACTGG	AAAGCGGCCGCCTGGACTTGCTTGAAGGAGAC
	PI5918	AAAGAATTCATGCCAGTACTTGACGTAACTGG	AAAGCGGCCGCCTGGACTTGCTTGAAGGAGAC
	PI6362	AAAGAATTCATGCTACCTAGTGATGCTACTCCAGTACTT	AAAGCGGCCGCCTGGACTTGCTTGAAGGAGAC
	PI8234	AAAGAATTCATGCCAGTACTTGACGTAACTGG	AAAGCGGCCGCCTGGACTTGCTTGAAGGAGAC
	PI8383	AAAGAATTCATGCCAGTACTTGACGTAACTGG	AAAGCGGCCGCCTGGACTTGCTTGAAGGAGAC
	PI9142	AAAGAATTCATGCTACCTAGTGATGCTACTCCAGTACTT	AAAGCGGCCGCCTGGACTTGCTTGAAGGAGAC
KTI-C	PI1410	AAAGAATTCATGCTTGTACTCCCTGAAGTTTATG	AAAGCGGCCGCCGCCTTGATGAACACAAATG
	PI4202	AAAGAATTCATGCTTGTACTCCCTGAAGTTTATGACC	AAAGCGGCCGCCGCCTTGATGAACACAAATG
	PI5446	AAACACGTGCGAAAAGAAAGTGATGGACTAGAAG	AAAGCGGCCGCACCAACCACAGGAATTTGTACA
PIN I	PI0234	AAAGAATTCATGCGAAAAGAATGTGATGG	AAAGCGGCCGCACCAACCACAGGCATTGATAC
	PI6013	AAAGAATTCAGAGATTTGATCAGTGATGGCAT	AAAGCGGCCGCACCCATACTGGGAGGAATTTG
PIN II	PI4434	AAAGAATTCATGAAGGCTTGCACTTTAGAATG	AAAGCGGCCGCCATTGCAGGGTACATATTTGCC
	PI6669	AAAGAATTCATGAAGGCTTGCACTTTAGAATG	AAAGCGGCCGCCATTGCAGGGTACATATTTGCC
PMEI	PI7531	AAAGAATTCATGGCGGGTAACGCC	AAAGCGGCCGCATGATTTGCAGCATATTGGTT

^a^Underlined sequences are the restriction enzyme recognition sites used for cloning in pPICZαA. GAATTC is recognized by *Eco*RI, CACGTG by *Pml*I and GCGGCCGC by *Not*I

### Expression of recombinant proteins

Recombinant *P. pastoris*—strains expressing a specific inhibitor were pre-cultured by inoculating 100 ml BMGY medium (1 % yeast extract, 2 % peptone, 100 mM potassium phosphate pH6, 1.34 % yeast nitrogen base, 0.0004 % biotin, 1 % glycerol) with a single colony. Cultures were incubated with continuous shaking at 28 °C for 24 h. Cells were harvested by centrifugation at 2,000×*g* for 10 min and re-suspended to an OD_600_ = 1 in 500 ml BMMY medium (1 % yeast extract, 2 % peptone, 100 mM potassium phosphate pH 6, 1.34 % yeast nitrogen base, 0.0004 % biotin, 0.5 % MeOH) to induce expression. *P. pastoris* cells were incubated for 72 h at 28 °C with continuous shaking. Every 24 h 100 % Methanol was added to a final concentration of 0.5 % to maintain induction. Cells were harvested by centrifugation for 10 min and 3,000×*g*. The supernatants were transferred to a new tube and subsequently used for protein purification.

### Purification of heterologous expressed inhibitors

Solid ammonium sulfate was added to the supernatant of the *Pichia pastoris* cell culture to a final concentration of 40 % and stirred for 30 min at room temperature (RT). After centrifugation at 10,000×*g* for 15 min at 4°, the pellet was dissolved in 5 ml binding buffer (0.02 M NaH_2_PO_4_, 1 M NaCl, 0.04 M imidazole, 5 % glycerol, pH 7.5). The protein solution was filtered through a syringe-attached 0.45 µm filter and loaded onto a 5 ml Protino^®^ Ni–NTA column (Macherey–Nagel, Düren, Germany) attached to an Äkta Prime Plus (GE Healthcare, Little Chalfont, UK) chromatographic system. The column was washed with 150 ml binding buffer to remove unbound proteins. The His-tagged fusion protein was eluted with 10 ml 0.02 M NaH_2_PO_4_, 1 M NaCl, 0.25 M imidazole, 5 % glycerol, pH 7.5. Fractions containing the fusion protein were combined and the elution buffer was replaced with 0.01 M Tris/HCl pH 7.5, 5 % glycerol using PD-10 desalting columns (GE Healthcare, Little Chalfont, UK) according to the manufacturer’s guidelines. Eluted fusion protein was concentrated tenfold by ultrafiltration using Amicon^®^ Ultra centrifugal filters (Millipore, Billerica, MS, USA). Protein concentration was estimated using Qubit^®^ Protein Assay (Invitrogen™, Carlsbad, USA) and BSA (bovine serum albumin) as standard. Proteins were snap frozen in liquid nitrogen and stored at −80 °C. Purification of fusion proteins was checked on SDS-PAGE using Anykd™ mini-protean^®^ TGX™ precast polyacrylamide gels (Bio-Rad, Hercules, California, USA) according to the manufactures guidelines. Western blot analysis was performed using 10 ng of purified protein and anti-c-myc antibody (Sigma Aldrich, St. Louis, MO, USA) following the protocol supplied with the antibody.

### Enzyme inhibition assays

All inhibition assays were performed with purified recombinant inhibitor protein on commercially available enzymes using colorimetric assays. Enzymes were pre-incubated without inhibitor protein (control) and with single and double amounts of inhibitor protein as specified below. After pre-incubation, the enzymatic reactions were started by adding the substrate. Absorbance was recorded in a microplate reader (Synergy 4, BioTek, Winooski, VT, USA) and used to calculate the residual enzyme activity. Enzyme inhibition was expressed as percentage of the enzyme activity in the controls (100 %). In a first round of experiments, three to five inhibitors were pooled in different molar ratios of inhibitor and enzyme and tested for enzyme inhibition. In case inhibitory activity of a pool on the tested enzyme was detected, the inhibitors composing the pool were re-tested individually and values for the half maximal inhibitory concentration (IC_50_) were calculated. IC50 values of active inhibitors were determined in triplicate by plotting percent initial activity against inhibitor concentrations from 0 to 8 µM in independent experiments.

Trypsin: Trypsin (EC 3.4.21.4) was purchased from Sigma Aldrich (St. Louis, USA). The trypsin inhibition assay was performed according to (Heibges et al. 2003b) with minor modifications using azocasein as substrate. Trypsin stock solution (5 mg/ml) was prepared in a buffer containing 50 mM Tris/HCl pH 7.5 and 20 mM CaCl_2_. 5 µg trypsin (0.21 µM) was pre-incubated for 10 min at RT either without (control), or with 0.5 and 1 µM purified inhibitor in 40 µl 100 mM Tris/HCl pH 7.5, 40 mM CaCl_2_. After adding 40 µl 2 % azocasein, the reactions were mixed by pipetting and incubated for 1 h at RT. Reactions were stopped by adding 80 µl 12 % TCA and incubation for 30 min at RT. Precipitate was removed by centrifugation and 100 µl supernatant was transferred to a 96-well plate. 50 µl 4 N NaOH were added and the absorbance at 440 nm was measured.

Elastase: Porcine pancreas elastase (PPE, EC 3.4.21.36) was supplied as part of the EnzChek^®^ Elastase Assay Kit (Invitrogen™, Carlsbad, USA). Human leukocyte elastase (HLE, EC 3.4.21.37) was purchased from Sigma Aldrich (St. Louis, USA). HLE was reconstituted with dH_2_O to 1U/µl. PPE and HLE were diluted to 0.2 U/ml in 100 µl assay buffer (100 mM Tris/HCl pH 8, 0.2 mM sodium azide) included in the EnzChek^®^ Elastase Assay Kit, and incubated for 10 min at RT with either none (control), 200 or 400 nM inhibitor protein. Elastase activity was determined according to the assay kit manufacturers’ guidelines including elastase inhibitor control reactions. Fluorescence intensity of inhibitor samples was corrected for background fluorescence determined from samples without enzyme.

Dipeptidylpeptidase 4 (DPP4): Screening for DPP4 (EC 3.4.14.5) inhibitors was performed using the DPP4 Drug Discovery Kit (Enzo^®^ Life Sciences, Farmingdale, New York, USA) according to the manufacturer‘s guidelines. DPP4 activity was detected using the chromogenic (H-Gly-Pro-pNA) substrate supplied with the kit. DPP4 was pre-incubated for 10 min at 37 °C with either none (control), 100 or 200 nM inhibitor protein. The assay was started by adding the substrate and the absorption at 405 nm was continuously read. A 405 nm values were plotted versus time until 15 min after addition of the substrate and a regression line was obtained. The slope was used to calculate DPP4 activity.

Factor IXa: Factor IXa (EC 3.4.21.22) was purchased from Abcam^®^ (Cambridge, UK) and was diluted to 90 ng/µl in assay buffer (50 mM Tris/HCl pH 7.4, 100 mM NaCl, 5 mM CaCl^2^, 40 % ethylene glycol). 20 µl of diluted enzyme solution was pre-incubated for 15 min at 37 °C with 200 µl assay buffer containing none (control), 50 or 100 nM protease inhibitor in a 96-well plate. The enzymatic reaction was initiated by adding 25 µl of 10 mM substrate solution (Pefachrome^®^ FIXa, Pentapharm, Basel, Switzerland) and increase in absorbance at 405 nm was recorded for 15 min at 37 °C. A linear regression was fitted to the linear proportion of the absorbance curve and the slope was calculated. The background slope determined from samples without enzymes was subtracted from enzyme and inhibitor samples.

Factor Xa: Factor Xa (EC 3.4.21.6) was purchased from Merck KGaA (Darmstadt, Germany) and was diluted to 5 ng/µl in assay buffer (50 mM Tris/HCl pH 7.4, 300 mM NaCl, 200 µg/ml BSA). 10 µl of diluted enzyme solution was incubated for 10 min at 37 °C in 80 µl assay buffer including either none (control), 10 or 20 nM inhibitor in a 96-well plate. 10 µl of 4 mM substrate solution (Pefachrome^®^ FXa, Pentapharm, Basel, Switzerland) was then added and the increase in absorbance at 405 nm for 5 min at 37 °C was recorded. A linear regression was fitted to the linear proportion of the absorbance curve and the slope was calculated. The background slope determined from samples without enzyme was subtracted from control and inhibitor samples.

Thrombin: Thrombin from human plasma (EC 3.4.21.5) was purchased from Sigma Aldrich (St. Louis, USA).Thrombin enzyme solution was diluted in assay buffer (10 mM HEPES pH 7.5, 150 mM NaCl, 200 µg/ml BSA) to 7.5 ng/µl. 10 µl diluted enzyme solution was incubated for 10 min at 37 °C either without (control) or with 20 and 40 nM purified inhibitor in 80 µl assay buffer in a 96-well plate. After adding 20 µl substrate solution (0.5 mg/ml N-Benzoyl-L-phenylalanyl-L-valyl-l-arginine-4-nitroanilide. Merck KGaA, Darmstadt, Germany) release of ρ-nitroanilide (ρNA) was monitored by the increase in absorbance at 405 nm for 5 min at 37 °C. Background absorption was determined from reaction mixtures without enzyme. A linear regression was fitted to the increase in absorbance and the enzyme activity was calculated from the slope. The slope of the background was subtracted from the slope of control measurements and the inhibitor samples.

β-Secretase (BACE-1): Screening for BACE (EC 3.4.23.46) inhibitors was performed using the BACE Inhibitor Screening assay kit (Cayman Chemical Company, Ann Arbor, MI, USA) according to the manufacturer’s instructions. BACE was pre-incubated for 15 min at 4 °C with either none (control), 100 or 200 nM inhibitor protein.

HIV1-Protease: Screening for HIV-1 protease (EC 3.4.23.16) inhibitors was performed using the Sensolyte^®^ 490 HIV1 Protease Assay Kit (Ana Spec, Fremont, CA, USA) according to the manufacturer’s guidelines. HIV-1 protease was purchased from Ana Spec (Fremont, CA, USA) and was diluted to 10 ng/µl (125 nM) with assay buffer complemented with Dithiothreitol (DTT) supplied with the kit. HIV-1 protease was pre-incubated for 15 min at room temperature with either none (control), 100 or 200 nM inhibitor protein.

Human Calpain-1: Screening for human calpain-1 (EC 3.4.22.52) inhibitors was performed using the Sensolyte^®^ AMC Calpain Activity Assay (Ana Spec, Fremont, CA, USA) according to the manufacturer’s protocol. Human calpain-1 (supplied with the kit) was pre-incubated for 5 min at 37 °C with none (control), 100 or 200 nM inhibitor protein.

Caspase-1: Screening for human Caspase-1 (EC 3.4.22.36) inhibitors was performed using the Caspase-1 Drug Discovery Kit (Enzo^®^ Life Sciences, Farmingdale, New York, USA) according to the manufacturer’s guidelines. Caspase-1 activity was detected using the chromogenic (AC-YVDA-pNA) substrate supplied with the kit. Caspase-1 was pre-incubated for 10 min at 30 °C with either none (control), 100 or 200 nM inhibitor protein. The assay was started by adding the substrate and the absorption at 405 nm was continuously read for 30 min. A 405 nm values were plotted versus time until 15 min after addition of the substrate and a regression line was obtained. The slope was used to calculate Caspase-1 activity.

Cathepsin K: Screening for Cathepsin K (EC 3.4.22.38) inhibitors was performed using the Cathepsin K Drug Discovery Kit (Enzo^®^ Life Sciences, Farmingdale, New York, USA) according to the manufacturer’s guidelines. Cathepsin K was pre-incubated for 30 min at 37 °C with either none, 100 or 200 nM inhibitor protein.

Matrix-Metalloproteinase-P9 (MMP-9): Screening for MMP-9 (EC 3.4.24.35) inhibitors was performed using the MMP-9 colorimetric drug discovery kit (Enzo^®^ Life Sciences, Farmingdale, New York, USA) according to the manufacturer’s guidelines. MMP-9 was pre-incubated for 30 min, at 37 °C with either none (control), 200 or 400 nM inhibitor protein.

5-Lipoxygenase (5-Lox): Lipoxygenase (EC 1.13.11.34) activity was assayed using the Lipoxygenase Inhibitor Screening Assay Kit (Cayman Chemical Company, Ann Arbor, MI, USA) according to the manufactures guidelines. 5-Lipoxygenase from *Solanum tuberosum* (potato) was purchased from Cayman Chemical Company (Ann Arbor, MI, USA). Linolenic acid was used as substrate. Residual 5-Lox activity was determined after pre-incubation for 15 min at 4 °C with either none (control), 200 or 400 nM protease inhibitor. Data analysis was performed according to the instructions supplied with the assay kit.

## Results

### Isolation of novel inhibitor variants from tuber cDNA libraries

**Fig. 1:**
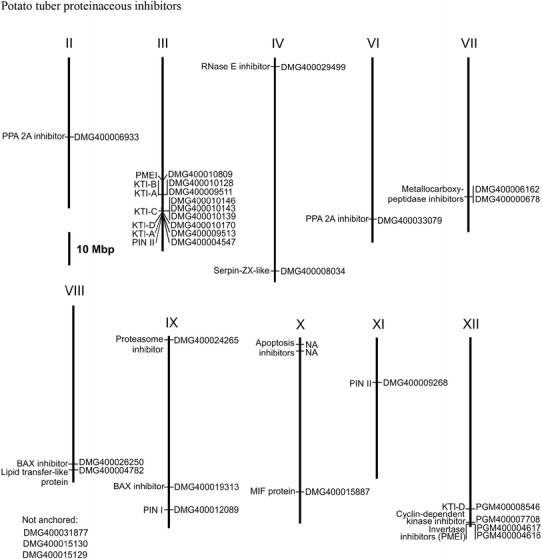
Physical map of genes encoding potato proteinaceous enzyme inhibitors. Genomic positions are according to the potato pseudomolecules (version 4.03) at http://potato.plantbiology.msu.edu/cgi-bin/gbrowse/potato/. Locus identifiers are shown to the right and names of encoded inhibitors are shown to the left of the chromosomes. Further details are shown in supplemental Table 1. NA: the locus is not annotated in the potato genome

In total 9,600 cDNA clones were randomly selected from small libraries prepared from mature tuber mRNA of ten potato genotypes (960 ESTs/cDNA library) and sequenced. One-hundred and seventy-five mostly full length cDNA clones encoding various inhibitor types were identified based on sequence similarity to known inhibitors in the GenBank core collection. After the removal of duplicate sequences, 120 unique inhibitor sequences remained, which matched to 31 loci in the potato genome (Fig. 1, supplemental Table 1). The deduced polypeptide sequence of 88 inhibitors (73 %) differed between 1 % and 18 % from inhibitor sequences present in the GenBank core collection. The nucleotide sequences fell into 19 structural groups with the largest group corresponding to Kunitz-type inhibitors A and B (supplemental Table 1). The Kunitz-type protease inhibitor (KTI) family accounted for 52 % of the inhibitor collection. The KTI family is divided into six structural groups (A, B, C, D, M and K) (Speranskaya et al. 2012), four of which were represented in the inhibitor collection (A, B, C and D). Fourteen KTIs assigned to group A (KTI-A), 33 in group B (KTI-B),12 in group C (KTI-C) and 4 in group D (KTI-D) matched to two, one, three and two loci, respectively, in the potato genome (PGSC 2011)(supplemental Table 1, Fig. 1).The second largest group corresponded to the potato protease inhibitor (PIN) family. PINs are divided in two structural groups. Accordingly, 9 and 8 cDNAs were classified as members of the subfamilies PIN I and PIN II, respectively. The 17 PINs matched to three loci. Nine sequences were assigned to pectin methylesterase inhibitors (PMEI) derived from three loci. Seven cDNAs each were identified as metallocarboxypeptidase inhibitors and defensins or Gamma-thionins. The remaining 17 cDNAs were classified in other inhibitor families like BAX, RNase E and putative phosphatase 2A (PPA 2A) inhibitors.

### Inhibitor heterologous expression and purification

**Fig. 2:**
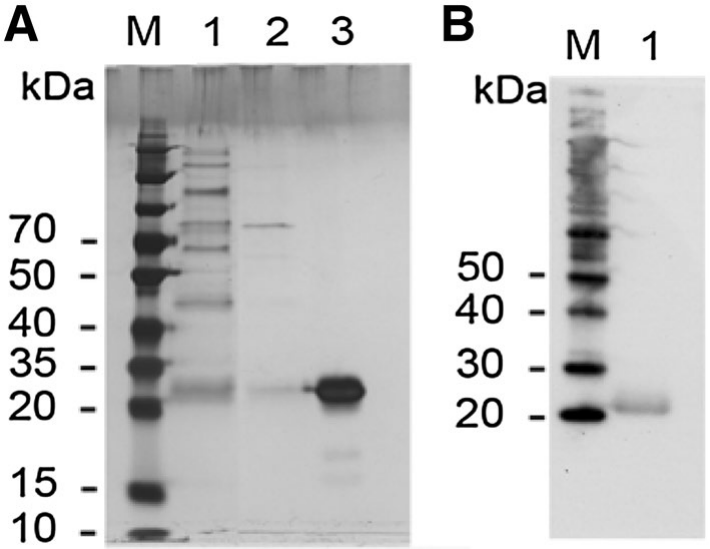
Purification of the PI1410 fusion protein from *P. pastoris* culture media by affinity chromatography. **a** Coomassie stained SDS-PAGE. Lane 1, *P. pastoris* culture media supernatant after 72 h induction of PI1410 expression by methanol. Lane 2, proteins precipitated with 40 % Ammonium sulfate from *P. pastoris* culture media. Lane 3, purified and concentrated PI1410 inhibitor protein. **b** Western blot analysis. Lane 1, purified and concentrated PI1410 inhibitor protein following incubation with anti-myc-tag antibody. *M* molecular weight standards

Twenty-nine inhibitors were selected for functional characterization of fusion proteins expressed in *Pichia pastoris* (supplemental Figure 1). The majority of the inhibitors were chosen based on previously described pharmacological properties of representatives of the KTI and PIN families. The major KTI structural groups A, B and C were represented by seven, twelve and three inhibitor variants, respectively. The PIN family was represented by two inhibitors each of the PIN I and PIN II sub-family. In addition to the KTI and PIN representatives, one PMEI, one RNase E and the unique ‘Cyclin dependent kinase inhibitor 2′ were selected. The sequences corresponding to the mature inhibitor polypeptides of 23 inhibitors (5 KTI-A, 10 KTI-B, 3 KTI-C, 2 PIN I, 2 PIN II, 1 PMEI) were successfully expressed in *Pichia pastoris*. Expression of seven inhibitors could not be detected in the *P. pastoris* system. Fusion proteins were purified from the culture medium by affinity chromatography. As an example, purification of the KTI-C inhibitor PI1410 is shown in Fig. 2. Following SDS-PAGE a faint band of 23 kDa was visible in the *P.pastoris* growth-media corresponding to the expected size of the fusion protein (Fig. 2a, Lane 1). Further purification by ammonium sulfate precipitation and affinity chromatography resulted in an enrichment of the 23 kDa protein band (Fig. 2a, Lane 3). The purified fusion protein was identified by Western blot analysis using a myc-tag antibody as probe (Fig. 2b, Lane 1). All 23 inhibitors were expressed in *P. pastoris* cells as described for PI1410 and expression was verified by Western blot analysis (data not shown). Inhibition assays were carried out using the purified inhibitor fusion protein.

### Protease and enzyme inhibition by recombinant inhibitor proteins

Purified inhibitor proteins were tested for in vitro inhibition of the serine proteases trypsin, elastase (pig pancreas, PPE, human leukocyte, HLE), dipeptidylpeptidase IV (DPP IV), factor IXa (FIXa), factor Xa (FXa) and thrombin. Four inhibitors (PI6033, PI0875, PI6362, PI0234) did not show any inhibitory activity on any of the enzymes tested (not shown). The serine proteases HLE, DPP IV, FIXa, FXa and thrombin, the cysteine proteases Calpain-1, Caspase-1 and the matrix-metallopeptidase-9 (MMP-9) were not affected by any of the tested inhibitors (not shown). Many inhibitors from the KTI, PIN and PMEI families are known to inhibit trypsin (Bauw et al. 2006; Heibges et al. 2003b; Jorgensen et al. 2006; Pouvreau et al. 2001). Trypsin inhibition among and within the inhibitor families was highly diverse, ranging from 0 to 87 % inhibition (Table 2). Among the KTIs, trypsin was inhibited primarily by members of subgroup B and percent inhibition varied from 16 % (PI8311) to 83 % (PI5918) (Table 2). KTI-A and KTI-C inhibitors had no or minor inhibitory effects on trypsin. Three PINs showed inhibitory properties comparable to KTI-B proteins (Table 2). The representative of the PMEI family (PI7531) showed only mild trypsin inhibition (10 %). Both PIN II inhibitors PI4434 and PI6669 inhibited the pig pancreas elastase with IC50 values of 0.67 µM and 0.97 µM, respectively (Table 2). Furthermore, the fusion proteins were tested for inhibition of the aspartyl proteases β-secretase (BACE) and HIV-1 protease. Inhibitors related to KTI-B and KTI-C subfamilies were responsible for BACE inhibition when tested individually. The KTI-B inhibitors PI8234, PI2112, PI5887 and PI4587 and the KTI-C inhibitor PI5446 strongly inhibited BACE with IC50 values between 0.36 µM and 0.81 µM (Table 2). HIV-1 protease was inhibited by the PIN I inhibitor PI6013 with an IC50 value of 0.67 µM (Table 2). The cysteine protease Cathepsin K was effectively inhibited by all three KTI-Cs tested (PI410, PI5446, PI4202) and by the PIN I inhibitor PI6013, which in addition inhibited the HIV-1 protease (Table 2). The KTI-C inhibitors with IC50 values between 0.02 and 0.09 µM were more effective than the PIN I inhibitor with an IC50 value of 0.77 µM. 5-Lipoxygenase was the only non-proteolytic enzyme tested. 5-Lipoxygenase was exclusively inhibited by all three KTI-C inhibitors PI1410, PI5446 and PI420 with IC50 values of 1.59 µM, 1.01 µM and 1.28 µM, respectively.

**Table 2:** In vitro inhibition of proteases and lipoxygenase by heterologous expressed potato tuber protease inhibitors

Inhibitor type	Inhibitor ID	Trypsin EC3.4.21.4^a^	Pig pancreas elastase EC3.4.21.36	BACE (β-secretase) EC3.4.23.46	Cathepsin K EC3.4.22.38	5-Lipoxygenase EC1.13.11.34	HIV-1 protease EC3.4.23.16
KTI-A	PI9070	9	–	–	–	–	–
KTI-A	PI4063	6	–	–	–	–	–
KTI-B	PI4435	62	–	–	–	–	–
KTI-B	PI4587	53	–	+ (IC50 0.81 µM)	–	–	–
KTI-B	PI8234	59	–	+ (IC50 0.4 µM)	–	–	–
KTI-B	PI2112	65	–	+ (IC50 0.36 µM)	–	–	–
KTI-B	PI2568	55	–	–	–	–	–
KTI-B	PI5887	38	–	+ (IC50 0.47 µM)	–	–	–
KTI-B	PI8383	61	–	–	–	–	–
KTI-B	PI5918	83	–	–	–	–	–
KTI-B	PI8311	16	–	–	–	–	–
KTI-B	PI9142	53	–	–	–	–	–
KTI-C	PI1410	0	–	–	+ (IC50 0.09 µM)	+ (IC50 1.59 µM)	–
KTI-C	PI5446	5	–	+ (IC50 0.72 µM)	+ (IC50 0.08 µM)	+ (IC50 1.01 µM)	–
KTI-C	PI4202	5	–	–	+ (IC50 0.02 µM)	+ (IC50 1.28 µM)	–
PIN I	PI6013	44	–	–	+ (IC50 0.77 µM)	–	+ (IC 50 0.67 µM)
PIN II	PI4434	43	+ (IC50 0.67 µM)	–	–	–	–
PIN II	PI6669	87	+ (IC50 0.97 µM)	–	–	–	–
PMEI	PI7531	10	–	–	–	–	–

^a^ % Inhibition at a molar ratio 2:1 of inhibitor: trypsin

## Discussion

Sequencing 9,600 randomly selected cDNA clones from mature potato tuber tissue of ten cultivars yielded 175 cDNA clones coding for various enzyme inhibitors according to sequence similarity. The overall transcript frequency of inhibitors was 1.8 %, which is in the same order of magnitude as previously observed in 1,600 cDNA clones from tubers of cultivars Provita and Saturna (Heibges et al. 2003a). The number of inhibitors isolated from individual cultivars varied widely, from three in cv Elfe to thirty-one in cv Solara, indicating large variation of inhibitor transcript levels, which could result from genotypic differences or different physiological states of the tubers used for library construction. Consistent with previous observations (Heibges et al. 2003a) the majority of all inhibitors slightly differed from sequences described in GenBank. The 120 unique nucleotide sequences matched to 31 loci on 10 of the 12 potato chromosomes. The number of coding loci could be smaller, as some inhibitors matched with similar high scores to more than one locus. This indicates that many sequence variants were allelic. In particular, the 59 KTI’s in groups A, B and C originated from only six loci, confirming the large allelic diversity of these genes.

To test whether structural variation is accompanied by functional diversification, we selected 29 representatives of various inhibitor classes for heterologous expression in the yeast *P. pastoris.* Twenty-three inhibitors could be expressed in soluble form and purified in sufficient quantity, mostly KTI’s and PIN’s. The recombinant proteins were evaluated for in vitro enzyme inhibitory functions. Four purified, soluble inhibitor proteins (PI6033, PI0875, PI6362, PI0234) did not show any biological activity, neither against trypsin, the most commonly used enzyme for protease inhibition assays, nor any of the other enzymes tested. These proteins might have folded incorrectly or lacked components required for activity or their activity spectrum is completely different from the one covered in our study.

The remaining nineteen proteins inhibited with different efficiency trypsin, the pharmacological important proteases pig pancreatic elastase, human β-secretase (BACE-1), human cathepsin K, and human HIV-1 protease, and potato 5-lipoxygenase. Trypsin and elastase are digestive proteases of insects that were previously shown to be targeted by plant PIs in the context of plant defense against herbivores (Hartl et al. 2011; Major and Constabel 2008; Turra et al. 2009). Among the tested KTI’s trypsin was effectively inhibited only by group B inhibitors, whereas group A and C KTI’s showed weak or no trypsin inhibition. This contrasts a previous study (Heibges et al. 2003b) where both A and B KTIs inhibited trypsin when the fusion proteins were expressed in the fission yeast *S. pombe*. The discrepancies may be the consequence of the different heterologous expression systems and tags used. The KTI-A inhibitor constructs expressed in *P. pastoris* might have been non-functional due to incorrect protein folding or lack of disulfide bridge formation. In addition to trypsin, four of ten biologically active KTI-B proteins inhibited BACE-1, demonstrating functional diversity within this group of allelic sequences. In agreement with previous functional studies (Glaczinski et al. 2002; Heibges et al. 2003b) trypsin inhibition by KTI’s in group C was low or even absent, which is likely due to a different substrate specificity. The three KTI’s in group C were biologically active since they all inhibited cathepsin K and potato 5-lipoxygenase. In addition, the KTI-C inhibitor PI5446 showed inhibitory properties on BACE-1. This functional difference presumably reflects the sequence diversity within the KTI-C family. The KTI group C proteins inhibited most effectively the cysteine protease cathepsin K, suggesting cysteine proteases as their main targets, although they show no homology to known cysteine protease inhibitor superfamilies (Bevec et al. 1996; Turk and Bode 1991). Further functions beyond protease inhibition are possible. Besides the newly identified inhibition of potato 5-lipoxygenase, KTI group C proteins have been found to inhibit in vitro soluble potato tuber invertase. KTI-C inhibitor PI4202 is identical to these putative invertase inhibitors characterized previously (Glaczinski et al. 2002).

Besides the serine protease trypsin, the potato proteinase inhibitor I (PIN I) PI6013 inhibited the cysteine protease cathepsin K and the aspartyl protease HIV-1. This PIN I protein seems to have multiple specificities. Previous studies had revealed high inter- and intra-specific structural and functional diversity among the PIN I family (Jorgensen et al. 2011; Turra et al. 2009). Native PIN I proteins have been suggested to occur as hexameric protein complexes in which every monomer represents a functional unit (van den Broek et al. 2004). PIN I monomers interact with the reactive center of a protease by a constrained loop protruding from the surface of the molecule (Mcphalen and James 1988; Mcphalen et al. 1985). Substrate specificity is supposed to be determined by the amino acid composition of the reactive loop. A lysine residue may interact with trypsin-like proteases while leucine or methionine at the same position in the reactive loop will bind to and inhibit proteases specific for hydrophobic side chains such as chymotrypsin (Jorgensen et al. 2011; van den Broek et al. 2004). PI6013 possesses an alanine residue at the corresponding position in the reactive loop, which might explain the different substrate specificities of this inhibitor.

Two representatives from the potato proteinase inhibitor family II (PIN II) inhibited trypsin and in addition elastase from pig pancreas (PPE). The activity of human leukocyte elastase was not affected. Members of the PIN II family are mainly found in the Solanaceae. Initially characterized in potato tubers they have been detected in leaves, flowers, fruits and phloem tissue of different Solanaceous species (Christeller and Laing 2005; Iwasaki et al. 1971; Miller et al. 2000; Pearce et al. 1993, 1988). Interestingly, PPE has been used as model system in several pharmacological screening assays for finding human leucocyte elastase (HLE) inhibitors. PIN II proteins function in planta presumably in defense reactions in response to the attack of herbivores and other pests (Hartl et al. 2010). The PIN II proteins PI4434 and PI6669 may act on insect gut proteases as part of constitutive defense mechanisms against herbivores in potato tubers and other tissues.

Diversity and abundance of plant protease inhibitors make them excellent sources for discovering novel enzyme inhibitors with specific pharmacological effects. Previous studies revealed several plant proteinase inhibitors acting on proteases involved in blood coagulation, fibrinolysis, inflammation or tumor cell proliferation [reviewed in (Oliva and Sampaio 2009) and (Fear et al. 2007)]. The therapeutic effect of a soybean derived Bowman–Birk inhibitor (BBI) in inflammatory diseases and cancer has been shown in several completed or ongoing human trials (Safavi and Rostami 2012). To the best of our knowledge, this is the first report of plant-derived Kunitz-type inhibitors with inhibitory activity on BACE-1 and cathepsin K, and a PIN I protein restricting HIV-1 protease activity. Human BACE-1 catalyzes the initial step in amyloidogenic metabolism of the large transmembrane amyloid precursor protein (APP) leading to the release of the amyloid β-peptide (Aβ) (Sinha and Lieberburg 1999). Aβ was identified as the main constituent of extracellular plaques in human brain which are characteristic for Alzheimer’s disease (Glenner and Wong 1984; Masters et al. 1985). Inhibition of BACE-1 could potentially reduce the extent of amyloid plaques and slow down or reverse the progression of the disease. Hence, the design of selective inhibitors of BACE-1 has been the target of intensive pharmaceutical development (Ghosh et al. 2008). Clinical effective BACE-1 inhibitors require high specificity and the ability to penetrate the blood–brain barrier and neuronal membranes. The KTI-B inhibitors of BACE-1 in our set exhibited higher substrate specificity compared to the KTI-C inhibitor, which qualifies them as lead structures for further research on drug development. The tested KTI-C inhibitors (PI5446, PI1410 and PI4202) were the most effective inhibitors of cathepsin K in our experiments. Cathepsin K is a lysosomal cysteine protease involved in osteoclast-mediated bone resorption. Inhibition of cathepsin K represents a potentially effective therapeutic approach for treating diseases characterized by excessive bone resorption, such as osteoporosis. KTI-C inhibitors showed a broader range of biological activity than KTI-B inhibitors, since members of this group inhibited 5-lipoxygenase in addition to BACE-1 and Cathepsin K. Nevertheless, the KTI-C inhibitors present potent starting points for protein engineering with the aim to increase substrate specificity. The same is true for the PINI inhibitor PI6013, which showed similar inhibitory properties on cathepsin K as well as HIV-protease, which is involved in virus proliferation (Sundquist and Kräusslich 2012).

The biological activity of potato KTIs is not limited to protease inhibition. Besides inhibition of invertase by specific members of the KTI-C family (Glaczinski et al. 2002), we demonstrated in this study that three KTI-C recombinant proteins inhibit potato 5-lipoxygenase. Plant lipoxygenases (LOX) are key enzymes in the biosynthesis of oxylipins, which play important roles in plant defense and development (Feussner and Wasternack 2002). The substrates of LOX are polyunsaturated fatty acids. The 5-LOX activity converts arachidonic acid in 5-Hydroperoxyeicosatetraenoic acid (5-HPETE), an intermediate in the biosynthesis of leukotrienes and other eicosanoids. The 5-LOX pathway is not only relevant for plant defense (Bostock et al. 1992) but also for inflammatory processes in humans leading to pathologies such as asthma and allergic reactions (Harizi and Gualde 2004). 5-LOX inhibitors have, therefore, been the targets of drug development (Williams and Spector 2009). The inhibition of LOX also opens up new possibilities for the in planta function of KTIs beyond protease inhibition. The new inhibitory functions discovered in this study might be just the tip of an iceberg, as our functional screens covered only a small fraction of enzymatic reactions. Screening recombinant potato inhibitors on additional classes of enzymes might reveal further biological activities.

In conclusion, we provide novel insights into the structural and functional diversity of proteinaceous inhibitors of potato tubers, which have potential for biotechnological applications. We identified inhibitors for proteases involved in severe human diseases, which may be used in drug development. Further studies of the mechanism of action and binding of natural inhibitors can provide leads for developing therapeutic agents against human diseases. The discovery of LOX inhibition by certain Kunitz-type inhibitors opens new possibilities for the in vivo functional role of this abundant class of tuber proteins.

## Electronic supplementary material

Below is the link to the electronic supplementary material.

Supplementary material 1 **Table** **1**. One-hundred and twenty potato tuber proteinaceous inhibitors: classification, annotation, GenBank accession, similarity to known inhibitors, position in the potato genome (XLSX 52 kb)

Supplementary material 2 **Fig. 1**. DNA sequences and primer sequences of 29 protease inhibitors selected for heterologous expression in *P. pastoris* (PDF 54 kb)
