# Supplementary material for: Novel in vitro inhibitory functions of potato tuber proteinaceous inhibitors
Source: Mol Genet Genomics. 2014 Sep 27;290(1):387–98. doi: 10.1007/s00438-014-0906-5 (PMC4309916; doi:10.1007/s00438-014-0906-5)
Supplement: Supplementary file 2 — Supplementary material 2 Fig. 1. DNA sequences and primer sequences of 29 protease inhibitors selected for heterologous expression in P. pastoris (PDF 54 kb) [file 438_2014_906_MOESM2_ESM.pdf]

Molecular and General Genomics

Novel in Vitro Inhibitory Functions of Potato Tuber Proteinaceous Inhibitors

Matthias Fischer[1], Markus Kuckenberg[1], Robin Kastilan[2], Jost Muth[2], Christiane Gebhardt[1]

[1] Max-Planck Institute for Plant Breeding Research, Carl von Linné Weg 10, 50829 Cologne, Germany

[2] Fraunhofer Institute for Molecular Biology and Applied Ecology, Forckenbeckstraße 6, 52074 Aachen, Germany

Corresponding author: C. Gebhardt, gebhardt@mpipz.mpg.de

Supplemental Figure 1: Nucleotide sequences and primers used for expression cloning of 29 potato tuber proteinaceous inhibitors. Borders of the expressed sequences are highlighted grey.

KTI-A type

PI8311

M031-140-1-E22-Eco	AAAGAATTCATGCTACCCAGTGCTAAGTCTGT
M032-140-1-E22-Not	AAAGCGGCCGCGTCTTCGACTTTCTCAAATTCGA

Cacaaatcaaacatgatgacgacgaagtgtttatttatcttatgtctgtgtttggttccctttgtggtgttttcatcaactttcacttcccaaaatcccattgacc
tacccagtgctaagtctgtcccggtactagacacgacgggtaaagaagttgaccctcgttggagttatcgtatggtttacactaaacggggtccctatg
gtggtgatatatacctagattactccccaggttcaactgccccttgtccagacggcgtgttccgttacggtcaggttggacctaagggtacacccgtca
gattgattacacctagtcattttggaccaggtgtgtatgtagatcaagagatcaacatccaattcgtcatttccaatgtagaaaagtgtggtagctataca
atttggaaagtcggaccttacgataatgaagatcgggtttcgtttttggagacgggccaacaaaatagcaagtcatgtttcaagattgtgaaatcacca
cgattattgggttacgaattaattacttgcgatggtgcattagtgggtactatgggccagcgtgtggcccttgtgagtaacttctcacttgatttcgaatttga
gaaagtcgaagactag

PI6033

M011-PI6033-Eco	AAAGAATTCATGGAATCTCCTGTACCTAAGCC
M012-PI6033-Not	AAAGCGGCCGCGACTTCCTGGAATAAGACGTCAA

tacacaaataaaatcaatatgatgaagtgtttatttttcttatgtctatgtttgtttcctattttggtgttttcatcaactttcacttcccaaaatcccatcaacctac
ccagtgaatctcctgtacctaagccggtacttgacacaaatggtaaaaaactgaatcctaattcgagttatcgcattatttccacattttggggtgcctta
ggtggtgatgtatacctaggaaagtccccaaattcagatgccccttgtccagatggcgtattccgttacaattccgatgttggacctagcggtacaccc
gttagattcattcctttatctacaaatatctttgaagatcaactacttaacatacaattcaatattccaacagtgaaattgtgtgttagttatacaatttggaaa
gtcggaaatctaaatacacatctatggactatgttgttggagacgggaggaaccatagggaaagcagatagcagctatttcaagattgttaaatcatc
aaaatttggttacaacttattgtattgccctattactcgccccccctattgtttgtccattttgtcgtgatgatgacttctgtgcaaaagtgggtgtagttattcaaa
atggaaaaaggcgtttggctcttgtcaacgaaaatcctcttgacgtcttattccaggaagtctag

PI6137

M011-PI6137-Eco AAAGAATTCATGGAATCTCCTGTACCTAAGCC
M040-PI6137-Not AAAGCGGCCGCGACTTCCTGGAATAAGACATCAAGA

atgatgaagtgtttatttttcttatgtttgtgtttgtttccaattttggtgttttcatcaactttcacttcccaaaatcccatcaacctacccagtgaatctcctgtacc
taagccggtacttgacacaaatggtaaaaaactcaatcctaattcgagttatcgcattatttccacattttggggtgccttaggtggtgatgtgtacctagg
aaagtccccaaattcagatgccccttgtccagatggtgtattccgttacaattccgatgttggacctaacggtacacccgttagattcattcctttatctac
aaatatctttgaagatcaactactcaacatacaattcaatattccaacagtgaaattgtgtgttagttatacaatttggaaagtcggtaatctaaatgcac
atctaaggacgatgttgttggagaccggaggaaccatagggcaagcagatagcagctatttcaagattgttaaatcatcaaaattgggttacaactta
ttgtattgccctatttctcgccattttctttgtccattttgtcgtgatgataacttctgtgcagaagtgggtgtagttattcaaaatggaaaaaggcgtttggctct
tgtcaacgaaaatcctcttgatgtcttattccaggaagtctag

PI9070

M011-PI6137-Eco AAAGAATTCATGGAATCTCCTGTACCTAAGCC
M040-PI6137-Not AAAGCGGCCGCGACTTCCTGGAATAAGACATCAAGA

atcaatatgatgaagtgtttatttttcttatgtttgtgtttgtttccaattttggtgttttcatcaactttcacttcccaaaatcccatcaacctaccccgtgaatctcc
tgtacctaagccggtacttgacacaaatggtaaaaaactcaatcctaattcgagttatcgcattatttccacattttggggtgccttaggtggtgatgtgta
cctaggaaagtccccaaattcagatgccccttgtccagatggtgtattccgttacaattcccatgttggacctaacggtacacccgttagattcattccttt
atctacaaatatctttgaagatcaactactcaacatacaattcaatattccaacagtgaaattgtgtgttagttatacaatttggaaagtcggtaatctaaa
tgcacatctaaggacgatgttgttggagaccggaggaaccatagggcaagcagatagcagctatttcaagattgttaaatcatcaaaattgggttac
aacttattgtattgccctatttctcgccattttctttgtccattttgtcgtgatgataacttctgtgcagaagtgggtgtagttattcaaaatggaaaaaggcgttt
ggctcttgtcaacgaaaatcctcttgatgtcttattccaggaagtctag

PI0875

M011-PI6137-Eco AAAGAATTCATGGAATCTCCTGTACCTAAGCC
M040-PI6137-Not AAAGCGGCCGCGACTTCCTGGAATAAGACATCAAGA

acacaaataattaaaatcaatatgatgaagtgtttatttttcttatgtttctgtttgtttcccattttggtgttttcatcaactttcacttcccaaaatcccatcaacc
tacccagtgaatctcctgtacctaagccggtacttgacacaaatggtaaaaaactcaatcctaattcgagttatcgcattatttccacattttggggtgcct
taggtggtgatgtgtacctaggaaagtccccaaattcagatgccccttgtccagatggtgtattccgttacaattccgatgttggacctaacggtacacc
cgttagattcattcctttatctacaaatatctttgaagatcaactactcaacatacaattcaatattccaacagtgaaattgtgtgttagttatacaatttgga
aagtcggtaatctaaatgcacatctaaggacgatgttgttggagaccggaggaaccatagggcaagcagatagcagctatttcaagattgttaaatc
atcaaaattgggttacaacttattgtattgccctatttctcgccattttctttgtccattttgtcgtgatgataacttctgtgcagaagtgggtgtagttattcaaa
atggaaaaaggcgtttggctcttggtcaacaaaatcctcttgatgtcttattccaggaagtctaa

PI2850

M011-PI6137-Eco	AAAGAATTCATGGAATCTCCTGTACCTAAGCC
M040-PI6137-Not	AAAGCGGCCGCGACTTCCTGGAATAAGACATCAAGA

Aataaaatcaatatgatgaagtgtttatttttcttatgtctgtgtttgtttcccattttggtattttcatcaactttcacttcccaaaatcccatcaacctacccagt
gaatctcctgtacctaagccggtacttgacacaaatggtaaaaaactgaatcctaattcgagttatcgcattatttccacattttggggtgcgttaggtggt
gatgtgtacctaggaaagtccccaaattcagatgccccttgtccagatggcatattccgttacaattccgatgttggacctagcggtacacccgttagat
tcattcctttatctacaaatatctttgaagatcaactacttaacatacaattcaatattccaacagtgaaattgtgtgttagttatacaatttggaaagtcgga
aatctaaatgcacagctaaggacgacgttgttggagacgggaggaaccataaggcaagcagatagcagctatttcaagattgttaaatcattaaaa
tttggttacaacttattgtattgccctattactccccctattactccccctattctttgtccattttgtcgtgatgataacttctgtgcaaaagtgggtgtagttattc
aaaatggaaaaaggcgtttggctcttgtcaacaaaaatcctcttgatgtcttattccaggaagtctag

PI4063

M041-141-1-p9_Eco	AAAGAATTCATGGATACTACTCCATGTACTCCAGT
M042-141-1-p9_Not	AAAGCGGCCGCGACTTCCTGGAAATAGACATCAAGA

Ttgtttcccattttggtgttttcatcaactttctcttcccaaaatcccattgacctacccactgatactactccatgtactccagtacatgacacaaatggtaat
gaacttaatcctaattcgagttatcgcattatttccactttttggggtgccttagggggtgatgtgtacctaggaaagtccccaaattcagatgccccttgtc
cagatggcgtattccgttacaattccgatgttggacctagcggtacacccgttagattcattcctaaatatggagcaaatatatgtgaggatcaactact
caacatacaattcaatattccaacagtgaaattgtgtgttagttatacaatttggaaagttggaaatctaaatgcacatccaaggacgatgttgttggag
acgggaggaaccatagggcaagcagatagcagctatttcaagattgctaaatcatcaaaatttggttacaacttattgtattgccctattactcgcccta
ttatttgtccattttgtcatgatgaaaacttctgtgcaaaaatgggagtagttattcaaaatggaaaaaagcgtttggctcttgtcaacgaaaatcctcttga
tgtctatttccaggaagtctag

KTI-B type

PI4435

M009-140-2-h11-Eco	AAAGAATTCATGCTACCTAGTGATGCTACTCCAGTACTT
M010-140-2-h11-Not	AAAGCGGCCGCCTGGACTTGCATGAAGGAGAC

aataaaattagtagcttaaacaagatgaagtgtttatttttgttatgtttgtgtttggttcccattgtggtgttttcatcaactttcacttcccaaaatcccattaac
ctacctagtgatgctactccagtacttgacgtaactggtaaagaacttgattctcgtttgagttatcgtattatttccacattttggggtgcgttaggtggtgat
gtgtacctaggtaagtccccaaactcagatgccccttgtgcaaatggcatattccgttacaattcggatgttggacctagcggtacacccgttagattca
ttggttcatctagtcattttggacaaggtatctttgaaaatgaactactcaacatccaatttgctatttcaacatcgaaattgtgtgttagttatacaatttgga
aagtgggagattacgatgcatctctagggacgatgttgttggagactggaggaaccataggtcaagcagatagcagttggttcaagattgttaaatc
atcacaacttggttacaacttattgtattgccctgttactagtacaatgatttgtccattttcctctgaagatcaattctgttcaaaagttggtgtagttcaccaa
aatggaaaaagacgtttggctcttgtcaaggacaatcctcttgatgtctccttcatgcaagtccagtaa

PI4587

M009-140-2-h11-Eco AAAGAATTCATGCTACCTAGTGATGCTACTCCAGTACTT
M023-144-3-i12-Not AAAGCGGCCGCCTGGACTTGCTTGAAGGAGAC

Acaaataaaattagtagcttaaacaagatgaagtgtttatttttgttatgtttgtgtttggttcccattgtggtgttttcatcaactttcacttccaaaaatcccatt aacctacctagtgatgctactccagtacttgacgtagctggtaaagaacttgattctcgtttgagttatcgtattatttccactttttggggtgcgttaggtggt gatgtgtacctaggtaagtccccaaattcagatgccccttgtgcaaatggcatattccgttacaattcggatgttggacctagcggtacacccgttagatt cattggttcatctagtcattttggacaaggtatctttgaaaatgaactactcaacatccaatttgctatttcaacatcgaaattgtgtgttagttatacaatttg gaaagtgggagattacgatgcatctctagggacgatgttgttggagactggaggaaccataggtcaagcagatagcagttggttcaagattgttaaa tcatcacgatttggttacaacttattgtattgccctgttacaattatttgtccatcttgccctgatgatcaattctgttcaaaagttggtgtagttcaccaaaatg gaaaaagacgtttggctcttgtcaaggacaatcctcttgatgtctccttcaagcaagtccagtaa

PI8234

M027-AAM10743-Eco AAAGAATTCATGCCAGTACTTGACGTAACTGG
M023-144-3-i12-Not AAAGCGGCCGCCTGGACTTGCTTGAAGGAGAC

cacaaataaaattagtagcttaaacaagatgaagtgtttatttttgttatgtttgtgtttggttcccattgtggtgttttcatcaactttcacttcccaaaatccca ttaacctacctagtgatgctactccagtacttgacgtaactggtaaagaacttgatcctcgtttgagttatcgtattatttccactttttggggtgcgttaggtg gtgatgtgtacctaggtaagtccccaaattcagatgccccttgtgcaaatggtgtattccgttacaattccgatgttggacctagcggtacacccgtcag attcattggttcatctagtcattttggacaaggtatctttgaagatgaactactcaacatccaattcgctatttcaacatcgaaaatgtgtgttagttatacaa tttgtaaagtgggagattacgatgcatctctagggacgatgttgttggagactggaggaaccataagtcaagcagatagcagttggttcaagattgtta aatcatcacaatttggttacaacttattgtattgccctgttactactacaatgactttgccattttcctctgatgatcaattctgtttaaaagttggtgtagttcac caaaatggaaaaagacgtttggctcttgtcaaggacaatcctcttgatgtctccttcaagcaagtccagtaa

PI2112

M027-AAM10743-Eco AAAGAATTCATGCCAGTACTTGACGTAACTGG
M023-144-3-i12-Not AAAGCGGCCGCCTGGACTTGCTTGAAGGAGAC

Acaaataaaattagtagcttaaacaagatgaagtgtttatttttgttatgtttgtgtttggttcccattgtggtgttttcatcaactttcacttcccaaaatcccatt aacctacctagtgatgctactccagtacttgacgtaactggtaaagaacttgatcctcgtttgagttatcgtattatttccactttttggggtgcgttaggtggt gatgtgtacctaggtaagtccccaaattcagatgccccttgtgcaaatggtgtattccgttacaattccgatgttggacctagcggtacacccgtcagatt cattggttcatctagtcattttggacaaggtatctttgaagatgaactactcaacatccaattcgctatttcaacatcgaaaatgtgtgttagttatacaattt ggaaagtgggagattacgatgcatctctagggacgatgttgttggagactggaggaaccataggtcaagcagatagcagttggttcaagattgttaa atcatcacaatttggttacaacttattgtattgccctgttactactacaatgactttgccattttcctctgatgatcaattctgtttaaaagttggtgtagttcacc aaaatggaaaaagacgtttggctcttgtcaaggacaatcctcttgatgtctccttcaagcaagtccagtaa

PI2568

M027-AAM10743-Eco AAAGAATTCATGCCAGTACTTGACGTAACTGG
M023-144-3-i12-Not AAAGCGGCCGCCTGGACTTGCTTGAAGGAGAC

Aaaattagtagcttaaacaagatgaagtgtttatttttgttatgtttgtgtttgtttcccattgtggtgttttcatcaactttcacttcccaaaatcccattaaccta
cctagtgatgctactccagtacttgacgtaactggtaaagaacttgatcctcgtttgagttatcgtattatttccactttttggggtgcgttaggtggtgatgtg
tacctaggtaagtccccaaattcagatgccccttgtgcaaatggtgtattccgttacaattccgatgttggacctagcggtacacccgtcagattcattgg
ttcatctagtcattttggacaaggtatctttgaagatgaactactcaacatccaattcgctatttcaacatcgaaaatgtgtgttagttatacaatttggaaa
gtgggagattacgatgcatctctagggacgatgttgttggagactggaggaaccataggtcaagcagatagcagttggttcaagattgttaaatcatc
acaatttggttacaacttattgtattgccctgttactactacaatgactttgccattttcctctgatgatcaattctgtttaaaagttggtgtagttcaccaaaatg
gaaaaagacgtttggctcttgtcaaggacaatcctcttgatgtctccttcaagcaagtccagtaa

PI5887

M027-AAM10743-Eco AAAGAATTCATGCCAGTACTTGACGTAACTGG
M023-144-3-i12-Not AAAGCGGCCGCCTGGACTTGCTTGAAGGAGAC

Acaaataaaattagtagctttaacaagatgaagtgtttatttttgttatgtttgtgtttggttcccattgtggtgttttcatcaactttcacttccgaaaatcccatt
aacctacctagtgatgctactccagtacttgacgtaactggtaaagaacttgatcctcgtttgagttatcgtattatttccactttttggggtgcgttaggtggt
gatgtgtacctaggtaagtccccaaatccagatgccccttgtgcaaatggtgtattccgttacaattcggatgttggacctagcggtacacccgttatatt
cattggttcatctagtcattttggaccgcatatcttcgaagatgaactactcaacatccaattcgctatttcaacatcgaaattgtgtgttagttatacaatttg
gaaagtgggagattacgatgcatctctagggacgatgttgttggagactggaggaaccataggtcaagcagatagcagttggttcaagattgttaaa
tcatcacgatttggttacaacttattgtattgccctgttacaattatttgtccatcttgccctgatgatcaattctgttcaaaagttggtgtagttcaccaaaatg
gaaaaagacgtttggctcttgtcaaggacaatcctcttgatgtctccttcaagcaagtccagtaa

PI8383

M027-AAM10743-Eco AAAGAATTCATGCCAGTACTTGACGTAACTGG
M023-144-3-i12-Not AAAGCGGCCGCCTGGACTTGCTTGAAGGAGAC

cacaaataaaattagtagctttaacaagatgaagtgtttatttttgttatgtttgtgtttggttcccattgtggtgttttcatcaactttcacttccgaaaatcccat
taacctacctagtgatgctactccagtacttgacgtaactggtaaagaacttgatcctcgtttgagttatcgtattatttccactttttggggtgcgttaggtgg
tgatgtgtacctaggtaagtccccaaattcagatgccccttgtgcaaatggtgtattccgttacaattcggatgttggacctagcggtacacccgttagat
tcattggttcatctagtcattttggaccgcatatcttcgaagatgaactactcaacatccaattcgctatttcaacatcgaaattgtgtgttagttatacaattt
ggaaagtgggagattacgatgcatctctagggacgatgttgttggagactggaggaaccataggtcaagcagatagcagttggttcaagattgttaa
atcatcacgatttggttacaacttattgtattgccctgttacaattatttgtccatcttgccctgatgatcaattctgttcaaaagttggtgtagttcaccaaaat
ggaaaaagacgtttggctcttgtcaaggacaatcctcttgatgtctccttcaagcaagtccagtaa

PI5918

M027-AAM10743-Eco AAAGAATTCATGCCAGTACTTGACGTAACTGG
M023-144-3-i12-Not AAAGCGGCCGCCTGGACTTGCTTGAAGGAGAC

Agctttaacaagatgaagtgtttatttttgttatgtttgtgtttggttcctattgtggtgttttcatcaactttcacttcccaaaatcccattaacctacctagtgatg
ctactccagtacttgacgtaactggtaaagaacttgatcctcgtttgagttatcgtattatttccattggtaggggtgcgttaggtggtgatgtgtacctaggt
aagtccccaaattcagatgccccttgtgcaaatggtgtattccgttacaattcggatgttggacctagcggtacacccgttagattcattggttcatctagt
cattttggaccgcatatcttcgaagatgaactactcaacatccaattcgctatttcaacatcgaaattgtgtgttagttatacaatttggaaagtgggagat
tacgatgcatctctagggacgatgttgttggagactggaggaaccataggtcaagcagatagcagttggttcaagattgttaaatcatcacaacttggt
tacaacttattgtattgtccattttcctctgatgatcaattctgtttaaaagttggtgtagttcaccaaaatggaaaaagacgtttggctcttgttaaggacaat
cctcttgatgtctccttcaagcaagtccagtaa

PI9142

M009-140-2-h11-Eco AAAGAATTCATGCTACCTAGTGATGCTACTCCAGTACTT
M023-144-3-i12-Not AAAGCGGCCGCCTGGACTTGCTTGAAGGAGAC

Acaaataaaattagtagcttaaacaagatgaagtgtttatttttgttatgtttgtgtttggttcccattgtggtgttttcatcaactttcacttccaaaaatcccatt
aacctacctagtgatgctactccagtacttgacgtagctggtaaagaacttgattctcgtttgagttatcgtattatttccactttttggggtgcgttaggtggt
gatgtgtacctaggtaagtccccaaattcagatgccccttgtgcaaatggcatattccgttacaattcggatgttggacctagcggtacacccgttatatt
cattggttcatctagtcattttggacaaggtatctttgaaaatgaactactcaacatccaatttgctatttcaacatcgaaattgtgtgttagttatacaatttg
gaaagtgggagattacgatgcatctctngggacgatgttgttggagactggaggaaccataggtcaagcagatagcagttggttcaagattgttaaa
tcatcacaatttggttacaacttattgtattgccctgttactagtacaatgagttgtccattttcctctgatgatcaattctgtttaanagttggtgtagttcacca
aaatggaaaaagacgtttggctcttgtcaaggacaatcctcttgatgtctccttcaagcaagtccagtaa

PI9007

M009-140-2-h11-Eco AAAGAATTCATGCTACCTAGTGATGCTACTCCAGTACTT
M023-144-3-i12-Not AAAGCGGCCGCCTGGACTTGCTTGAAGGAGAC

Acaaataaaattagtagcttaaacaagatgaagtgtttatttttgttatgtttgtgtttggttcccattgtggtgttttcatcaactttcacttccaaaaatcccatt
aacctacctagtgatgctactccagtacttgacgtagctggtaaagaacttgattctcgtttgagttatcgtattatttccactttttggggtgcgttaggtggt
gatgtgtacctaggtaagtccccaaattcagatgccccttgtgcaaatggcatattccgttacaattcggatgttggacctagcggtacacccgttagatt
cattggttcatctagtcattttggacaaggtatcttttaaaatgaactactcaacatccaatttgctatttcaacatcgaaattgtgtgttagttatacaatttgg
aaagtgggagattacgatgcatctctagggacgatgttgttggagactggaggaaccataggtcaagcagatagcagttggttcaagattgttaaat
catcacaatttggttacaacttattgtattgccctgttactagtacaatgagttgtccattttcctctgatgatcaattctgtttaaaagttggtgtagttcaccaa
aatggaaaaagacgtttggctcttgtcaaggacaatcctcttgatgtctccttcaagcaagtccagtaa

PI9234

M009-140-2-h11-Eco AAAGAATTCATGCTACCTAGTGATGCTACTCCAGTACTT
M023-144-3-i12-Not AAAGCGGCCGCCTGGACTTGCTTGAAGGAGAC

Aacaagatgaagtgtttatttttgttatgtttgtgtttggttcccattgtggtgttttcatcaactttcacttccaaaaatcccattaacctacctagtgatgctactccagtacttgacgtagctggtaaagaacttgattctcgtttgagttatcgtattatttccactttttggggtgcgttaggtggtgatgtgtacctaggtaagtccccaaattcagatgccccttgtgcaaatggcatattccgttacaattcggatgttggacctagcggtacacccgttagattcattggttcatctagtcattttggacaaggtatctttgaaaatgaactactcaacatccaatttgctatttcaacatcgaaattgtgtgttagttatacaatttggaaagtgggagattacgatgcatctctagggacgatgttgttggagactggaggaaccataggtcaagcagatagcagttggttcaagattgttaaatcatcacaatttggttacaacttattgtattgccctgttactagtacaatgagttgtccattttcctctgatgatcaattctgtttaaaagttggtgtagttcaccaaaatggaaaaagacgtttggctcttgtcaaggacaatcctcttgatgtctccttcaagcaagtccagtaa

PI6362

M009-140-2-h11-Eco AAAGAATTCATGCTACCTAGTGATGCTACTCCAGTACTT
M023-144-3-i12-Not AAAGCGGCCGCCTGGACTTGCTTGAAGGAGAC

cttaaacaagatgaagtgtttatttttgtatgtttgtgtttggttcccattgtggtgttttcatcaactttcacttccaaaaatcccattaacctacctagtgatgctactccagtacttgacgtagctggtaaagaacttgattctcgtttgagttatcgtattatttccactttttggggtgcgttaggtggtgatgtgtacctaggtaagtccccaaattcagatgccccttgtgcaaatggcatattccgttacaattcggatgttggacctagcggtacacccgttagattcattggttcatctagtcattttggacaaggtatctttgaaaatgaactactcaacatccaatttgctatttcaacatcgaaattgtgtgttagttatacaatttggaaagtgggagattacgatgcatctctagggacgatgttgttggagactggaggaaccataggtcaagcagatagcagttggttcaagattgttaaatcatcacaatttggttacaacttattgtattgccctgttactagtacaatgagttgtccattttcctctgatgatcaattctgtttaaaagttggtgtagttcaccaaaatggaaaaagacgtttggctcttgtcaaggacaatcctcttgatgtctccttcaagcaagtccagtaa

KTI-C type

PI1410

M033-140-1-k03-eco AAAGAATTCATGCTTGTACTCCCTGAAGTTTATG
M008-139-2-c11-Not AAAGCGGCCGCCGCCTTGATGAACACAAATG

Atgaagtcgattaatattttgagtttcctcttgctttcaagtaccctctctttggttgcctttgctcgatctttcacttctgagaatccaattgtcctccccacaacttgtcatgatgatgataatcttgtactccctgaagtttatgaccaagatggcaatccgctgaggattggtgagaggtacattattaagaatcctctcctcggggccggagccgtatacttggacaatattggaaaccttcaatgcccaaacgccgtgttgcagcacatgtcaattccccaatttttgggaaaaggcacgcccgtcgtgttcattcgtaagtcggagtcggattatggtgatgtggtgcgtctaatgactgctgtttatatcaagttctttgttaaaacaacaaagttgtgtgttgacgaaactgtttggaaagttaataatgaacagttggtggtaactggtggtaacgtaggaaatgaaaacgacatcttcaagatcaagaaaactgacttggtgatacgaggtatgaaaaatgtatacaagttactgcattgtccctctcatcttgagtgcaaaaatatcggcagcaactttaaaaatggatatcctcgtctggtgactgtcaatgacgaanaggactttattccatttgtgttcatcaaggcgtag

PI5446

M007-139-2-c11-Eco	AAAGAATTCATGCTTGTACTCCCTGAAGTTTATGACC
M008-139-2-c11-Not	AAAGCGGCCGCCGCCTTGATGAACACAAATG

Gtcgattaatattttgagtttcctcttgctttcaagtaccctctctttggttgcctttgctcgatctttcacttctgagaatccaattgtcctgcccacaacttgtcatgatgatgataatcttgtactccctgaagtttatgaccaagatggcaatccgctgaggattggtgagaggtacattattaagaatcctctcctcggggccggagccgtatacttggacaatattggaaaccttcaatgcccaaacgccgtgttgcagcacatgtcgattccccaatttttgggaaaaggcacgcccgtcatgttcgttcgtaagtcggagtcggagtcggattatggtgatgtggtgcgtctaatgactgctgtttatatcaagttctttgttaaaacaacaagattttgtgttaaagaaactgtttggaaagttaatgatgaacagttggtggtaactggtggtaacgtaggaaatgaaaacgacatcttcaagattatgaaaactgacttggtgatacgaggtatgaaaaatgtatacaagttactgcattgtccctctcatcttcagtgcaaaaatatcggcagcaactttaaaaatggatatcctcgtctggtgactgtcgatggtgataaggactttattccatttgtgttcatcaaggcgtaa

PI4202

M007-139-2-c11-Eco	AAAGAATTCATGCTTGTACTCCCTGAAGTTTATGACC
M008-139-2-c11-Not	AAAGCGGCCGCCGCCTTGATGAACACAAATG

Tcgattaatattttgagtttcctttgctttcaagtaccctctctttggttgcctttgctcgatctttcacttctgagaatccaattgtcctccccacaacttgtcatgataatcttgtactccctgaagtttatgaccaagatggcaatccgctgaggattggtgagaggtacattattaagaatcctctcctcggggccggagccgtatacttggacaatattggaaaccttcaatgcccaaacgccgtgttacagcacatgtcgattccccaatttttgggaaaaggcacgcccgtcatgttcgttcgtaagtcggagtcggatgatggtgatgtggtgcgtctaatgactgctgtttatatcaagttctttgttaaaacaacaaagttgtgtgttgacgaaactgtttggaaagttaatgatgaacagttggtggtaactggtggtaacgtaggaaatgaaaacgacatcttcaagatcaagaaaactgacttggtgatacgaggtatgaaaaatgtatacaagttactgcattgtccctctcatcttcagtgcaaaaatatcggcagcaactttaaaaatggatatcctcgtctggtgactgtcgatgacgataaggactttattccatttgtgttcatcaaggcgtag

PIN I

PI0234

M015-140-2-c2-Eco	AAAGAATTCATGCGAAAAGAATGTGATGG
M016-140-2-c2-Not	AAAGCGGCCGCACCAACCACAGGCATTGATAC

Atggagtcaaagtttgctcacatcattgttttctttcttcttgcaacttcctttgaaactctcttggcacgaaaagaatgtgatggaccagaagtcatagaacttctaaaggaatttgaatgcaatggaaaacaaaggtggccagaacttattggtgtaccagcacagtatgctaagggaataattgagaaggaaaatacacttatagctgatgttcagataatactgaatggttctccagtcacagctgattttagttgtaatcgagttcgtattgctgtaaacatcttggattatgctgtatcaatgcctgtggttggttaa

PI6013

M045-PI6013-EcoRI	AAAGAATTCAGAGATTTGATCAGTGATGGCAT
M046-PI6013-NotI	AAAGCGGCCGCACCCATACTGGGAGGAATTTG

Aaaatggagggaaagagtatgctcaagttatctcatgtgcttgctttcttgcttcttgcatcactttttcaatcactgatggcaagagatttgatcagtgatggcatagaagtacttcaacttccagtggaaaatgaaggcgaatctgtattttgtccaggtaagctatcatggcctgaacttgttgggatgccagcaggatatgcaaaagcaataattgagaaggaaaattccatagttcatgatgttaggcttctacttcctggtatggttaagccagctaattatgtttgtggtagagttttctggttgttgaccttaaactcattgttcaaattcctcccagtatgggttag

PIN II

PI4434

M028-AAO88244-Eco	AAAGAATTCATGAAGGCTTGCACTTTAGAATG
M029-AAO88244-Not	AAAGCGGCCGCCATTGCAGGGTACATATTTGCC

Ttatccatcatggctgttcacaaggaagttaatttcgttgcttacctactaattgttcttggattattggtacttgtaagcgcgatggagcatgttgatgcgaa
ggcttgcactttagaatgtggtaatcttgggtttgggatatgcccacgttcagaaggaagtccggaaaatcgcatatgcaccaactgttgtgcaggttat
aaaggttgcaattattatagtgcaaatggggctttcatttgcgaaggagaatctgacccaaaaaacccaaatgtttgcccccgaaattgtgatacaaa
tattgcctattcaaaatgtctccgttcagaaggaaaatcgctaatttatcccaccgaatgtaccacatgctgcacagggtacaagggttgctactatttcg
gtaaaaatggcaagtttgtatgtgaaggagagagtgatgagcccaaggcaaatatgtaccctgcaatgtga

PI6669

M028-AAO88244-Eco	AAAGAATTCATGAAGGCTTGCACTTTAGAATG
M029-AAO88244-Not	AAAGCGGCCGCCATTGCAGGGTACATATTTGCC

Ttatccatcatggctgttcacaaggaagttaatttcgttgcttacctactaattgttcttggattattggtacttgtaagcgcgatggagcatgttgatgcgaa
ggcttgcactttagaatgtggtaatcttgggtttgggatatgcccacgttcagaaggaagtccggaaaaccgcatatgcaccaactgttgtgcaggttat
aaaggttgcaattattatagtgcaaatggggctttcatttgcgaaggagaatctgacccaaaaaacccaaatgtttgcccccgaaattgtgatacaaa
tattgcctattcaaaatgtctccgttcagaaggaaaatcgctaatttatcccaccgaatgtaccacatgctgcacagggtacaagggttgctactatttcg
gtaaaaatggcaagtttgtatgtgaaggagagagtgatgagcccaaggcaaatatgtaccctgcaatgtga

Pectin metylesterase inhibitor

PI7531

M025-136-2-f19-Eco	AAAGAATTCATGGCGGGTAACGCC
M026-136-2-f19-Not	AAAGCGGCCGCATGATTTGCAGCATATTGGTT

atggccgcagcgaaactgttatttctcgcagcacttttcctctctctctcctacctctccggcgcggcgggtaacgccgcagcctctaccagtttcataaa
aagctcttgtaaatccactacttttcccgatgtatgcgttgcttccctttccggttacgcacaagccattaaaaacagccaattgcagctggtgaaaacg
gctctgtccgtgagcctagataaagccgaatcgacaaaaggattcgttagcaagctgttgaaattcaagggattgaaaccgagagagtatgcagct
atcaaggattgcgtggaggaaacgaatgatagtgtagatcggcttagcagatctgtgaatgagctgaaaggtttggatcgtaaccatggaaagcc
gatttccagtggcatatgagtaacgtagagacatgggtaagtgctgctattacagatgagaatacttgcactgatggatttgccggccgagctttgaat
ggtaaaattaaggcttccattagaagtcacatagctaatctagctcaggttactagcaatgcactggccttaatcaaccaatatgctgcaaatcattga

Cyclin dependent kinase inhibitor 2

PI6203

M034-139-2-K16-PmI	AAACACGTGATGGGCAAGTATATGAGGAAGAG
M035-139-2-K16-NotI	AAAGCGGCCGCACGATCTACTTTCACCCATTTGTAA

Ggtgaaatgggcaagtatatgaggaagaggaagacaaaaactactggtgaagttgttgctgttttggatgtaagtcctttgggtgttcgtacaagagc
taaaaccctagctttgaagcaattgcagaaatcgagctccggcggcggtgacagtggtgatgtgggtgggggatgttatctgcagctgaggagcag
gaggctggagaaacccgtggtgggttttgaaaggaaaagacggaaacaccctttgaaggaatcgaaaagacagaatcggagtttgagggtgag
gaagatgaaggggcaatcttggaattctgggtctgttgagggtgaagaggagaagaagggagaagttcatgtacaagaaaatcagaaggaaatt
gacaacaatggcagctttgaaggtgaaaacttgctggaatttgatggtagagagaggaccaccagggagagcacaccttgcaatttgatcaggga
cccggataacataccaacccctggttccagtacaagacctaacaatgctagtgaagctaacggcagagagccaacttcagcgcaaagaattatc
ccaacaactcatgaaatgaatgacttctttgctggtacagaagagaagcagcagaaacaattcattgagaagtacaactttgatccggtgaacgac
aagcccctccctggacgttacaaatgggtgaaagtagatcgttag

RNase E inhibitor

PI1479

M036-142-2-L4-Eco	AAAGAATTCATGGCCTTGGTCACCACT
M037-142-2-L4-Not	AAAGCGGCCGCAACACATAGCTCTATTTTAGAAATCAGAA

cgtaggatggccttggtcaccactgctgaagtttgcgatgcaaatccacagcttattgtgagtggtgaactacgggcactgcagccaatcttcaaaat
atatggcaggcgccaagtcttctctggacctgttgtcactctgaaagtattcgaagacaatgttttggttcgtgagtttctcgaggagaaaggtaacggt
agagttcttgtagtcgacgggggtggtagtctaagatgtgcaattttgggtggcaaccctgtagtacaagctcaaaacaatggatgggctgggatcgt
agtaaatggctgtgtaagggacgtggacgaaatcaatggctgtgatatcggagtcagagctctggcttcacatccaatgaaagccaataagaaag
gtatcggagagaagcacgttcccataaccattgccgggactagaatctgcgatggtgagtggctttatgcagataccgatggcattctgatttctaaaa
tagagctatgtgtttga
